# Super-resolution microscopy reveals how histone tail acetylation affects DNA compaction within nucleosomes *in vivo*

**DOI:** 10.1093/nar/gkz593

**Published:** 2019-07-09

**Authors:** Jason Otterstrom, Alvaro Castells-Garcia, Chiara Vicario, Pablo A Gomez-Garcia, Maria Pia Cosma, Melike Lakadamyali

**Affiliations:** 1 ICFO-Institute of Photonic Sciences, Barcelona Institute of Science and Technology, Barcelona; 2 Centre for Genomic Regulation (CRG), The Barcelona Institute of Science and Technology, Dr. Aiguader 88, Barcelona 08003, Spain; 3 Universitat Pompeu Fabra (UPF), Barcelona, Spain; 4 ICREA, Pg. Lluis Companys 23, Barcelona 08010, Spain; 5 Guangzhou Regenerative Medicine and Health Guangdong Laboratory (GRMH-GDL), Guangzhou 510005, China; 6 Key Laboratory of Regenerative Biology and Guangdong Provincial Key Laboratory of Stem Cells and Regenerative Medicine, Guangzhou Institutes of Biomedicine and Health, Chinese Academy of Science, Guangzhou 510530, China; 7 Perelman School of Medicine, Department of Physiology, University of Pennsylvania, Clinical Research Building, 415 Curie Boulevard, Philadelphia, PA 19104, USA

## Abstract

Chromatin organization is crucial for regulating gene expression. Previously, we showed that nucleosomes form groups, termed clutches. Clutch size correlated with the pluripotency grade of mouse embryonic stem cells and human induced pluripotent stem cells. Recently, it was also shown that regions of the chromatin containing activating epigenetic marks were composed of small and dispersed chromatin nanodomains with lower DNA density compared to the larger silenced domains. Overall, these results suggest that clutch size may regulate DNA packing density and gene activity. To directly test this model, we carried out 3D, two-color super-resolution microscopy of histones and DNA with and without increased histone tail acetylation. Our results showed that lower percentage of DNA was associated with nucleosome clutches in hyperacetylated cells. We further showed that the radius and compaction level of clutch-associated DNA decreased in hyperacetylated cells, especially in regions containing several neighboring clutches. Importantly, this change was independent of clutch size but dependent on the acetylation state of the clutch. Our results directly link the epigenetic state of nucleosome clutches to their DNA packing density. Our results further provide *in vivo* support to previous *in vitro* models that showed a disruption of nucleosome-DNA interactions upon hyperacetylation.

## INTRODUCTION

Each chromosome of interphase, eukaryotic nuclei occupies a specific nuclear area organized in chromosomal territories (CT) (1,2). At the smallest of chromatin length scales, 146 bp of DNA wrap around an octamer of histone proteins, forming the nucleosome. Nucleosomes arrange as beads on a string along the DNA forming a fiber that is roughly 10 nm in diameter. Through the linker histone H1, the 10 nm fiber further compacts into higher order structures (3). *In vitro* reconstitution studies showed that nucleosomes form a very ordered zig-zag or solenoid-like organization compacting chromatin into a 30 nm fiber. However, the existence of this 30 nm fiber *in vivo* has been long debated and how the chromatin compacts and folds into higher order structures remains unclear. Several recent studies suggested that the organization of nucleosomes and higher order chromatin folding is much more heterogeneous than the regular 30 nm fiber (4–11). The emerging picture suggests that chromatin is largely comprised of 10 nm fibers with different levels of compaction. In line with these recent studies, our previous work using super-resolution microscopy demonstrated that nucleosomes form heterogeneous groups, termed nucleosome clutches (8). The size of nucleosome clutches was lower in cells having more open chromatin, including embryonic stem cells and induced pluripotent stem cells (iPSCs).

Chromatin compaction and folding is further shaped by histone post-translational modifications. The core histones, and in particular H3 and H4, have N-terminal protein tails protruding from the nucleosomes, which can be covalently modified at different residues (12,13). The tails can be methylated, acetylated, phosphorylated or ubiquitinated, among other modifications. The combination of these different modifications has been defined as ‘histone code’ (14). The histone code of nucleosomes positioned at promoter regions biases DNA accessibility to transcription factors and transcription machinery, and is an important regulator of gene activation and repression (15). Specifically, lysine acetylation neutralizes lysine positive charges. This neutralization results in a reduction of the electrostatic attraction between the histone tails and the negative charged DNA. It has thus been postulated (16,17) that histone acetylation results in a more ‘open’ chromatin fiber with the DNA more loosely attached to the nucleosomes. In addition, all-atom molecular dynamics simulations suggested that acetylation can disrupt inter-nucleosome interactions leading to unfolding and de-compaction of the chromatin fiber (18). This chromatin opening leads to an increased accessibility of the transcription factors and RNA Polymerase II holoenzyme and therefore to transcription activation (16,17). While previous work using *in vitro* reconstituted chromatin and all-atom molecular dynamics simulations lead to a molecular model of acetylation-induced chromatin fiber decompaction, how acetylation impacts chromatin structure *in vivo* is less clear.

We previously showed that in cells treated with the histone deacetylase (HDAC) inhibitor tricostatin A (TSA), which leads to histone tail hyperacetylation, the nucleosome clutches were smaller compared to untreated cells (8). Further, recent work showed a correlation between the size of chromatin domains, epigenetic modifications of these domains and DNA packing density within the domains (19). For example, regions with silencing epigenetic marks had larger domains and higher DNA packing density than regions with activating epigenetic marks. However, whether smaller clutches pack less DNA simply because they contain fewer nucleosomes (i.e. lower nucleosome occupancy) or whether the epigenetic modifications of the nucleosomes within a clutch impacts its DNA packing density is unclear.

To directly address these questions and move beyond correlations, we visualized histones together with DNA in control cells and cells treated with TSA using two-color, 3D super-resolution microscopy. We further carried out highly quantitative spatial analysis of DNA and histone co-organization in control and TSA-treated cells. This quantitative spatial analysis showed that a larger percentage of DNA was free of histones in fibroblasts with increased histone tail acetylation, suggesting a decrease in nucleosome occupancy. We further identified what we refer to as a ‘clutch’ DNA present within a small nanoscale area around the nucleosome clutches, whose compaction was influenced by the nucleosome clutch itself. This clutch DNA occupied a smaller area and was less dense in the TSA-treated cells. To directly determine whether DNA is less loosely packed around smaller clutches regardless of their histone modifications, we analyzed DNA packing density as a function of clutch size in control cells and TSA-treated cells. Interestingly, the packing density of DNA within nucleosome clutches was independent of the clutch size but the packing density was always lower in hyperacetylated clutches compared to control clutches. These results suggest that it is the acetylation state and not the clutch size that determines how tightly DNA is wrapped and compacted by nucleosomes within a clutch. Finally, we observed that the presence of nearby clutches greatly influenced the level of DNA compaction within a clutch. Combined together, our results unraveled that histone acetylation impacts DNA compaction through multifaceted mechanisms including decreased nucleosome occupancy and looser packing of DNA by nucleosome clutches with acetylated histone tails in particular in nucleosome-clutch-rich regions. Overall, our results quantify the structural reorganization and DNA decompaction induced by histone tail acetylation at the nanoscale level *in vivo*, complementing the molecular understanding previously inferred through *in vitro* experiments and mesoscale modeling.

## MATERIALS AND METHOD

### Cell culture and sample preparation

Human Fibroblasts (hFb) (BJ, Skin Fibroblast, American Type Culture Collection, ATCC CRL-2522) were cultured in DMEM (#41965062, Gibco) supplemented with 10% FBS (#10270106, Gibco), 1× non-essential amino acids (#11140050, Gibco), 1× Penicillin/Streptomycin (#15140122, Gibco) and 1× GlutaMax (#35050061, Gibco). For DNA labeling experiments, cells were cultured with 5 μM ethynil-deoxy-cytdine (EdC) (#T511307, Sigma-Aldrich) for 96 h previous to fixation. For hyperacetylation experiments, hFb were treated with 300 nM of TrychostatinA (TSA) (#T8552 Sigma-Aldrich) in complete growth medium supplemented with 5 μM EdC during the final 24 h of the 96 h EdC incubation before fixation. When using fiduciary markers for drift correction and 3D overlap, growth media with EdC was supplemented with 1:800 dilution of 160 nm amino yellow beads (#AFP-0252-2, Spherotech) for the final 1 h prior to fixation to permit internalization of the beads into the cells prior to fixation.

### Cell cycle analysis

hFb were grown in growth media supplemented with EdC for 96 h. The cells were collected and were resuspended gently with ethanol 70% at –20C. The cells were pelleted, washed and resuspended in propidium iodide (Molecular Probes, #P-1304) 0.03 mg/ml, Sodium Citrate 1.1 mM and RNAse A (Sigma, #R-5503) 0.3 mg/ml in PBS at 4°C overnight. Flow cytometry analysis was performed in a FACSCalibur (BD Biosciences). Cell cycle analysis was performed using the ModFit program.

### Staining for STORM

For imaging experiments, cells were plated on eight-well Lab-tek #1 borosilicate chambers (#155411, Nunc) at a seeding density of 20 000–30 000 per well for immunostaining experiments; and at a seeding density of 5000–10 000 cells per well for DNA labeling experiment to allow cell proliferation and hence EdC incorporation to the genome. The cells were fixed with 4% PFA (#43368, Alfa Aesar) diluted in PBS for 15 min at room temperature (RT). Then, they were permeabilized with 0.3% (v/v) Triton X-100 (#327371000, AcrosOrganics) in PBS for 15 min at room temperature. Cells were then blocked using 10% BSA (#9048468 Fisher Scientific) (w/v), 0.01% (v/v) Triton X-100 in PBS. Cells were incubated overnight with the rabbit polyclonal anti-H2B (Abcam, #1790) primary antibody diluted 1:50 in blocking buffer, at 4°C with gentle rocking. Finally, cells were washed three times with blocking buffer, then incubated with 1:50 secondary antibody for STORM Imaging (see below) for 1 h at room temperature. For PAINT labeling, we used the Ultivue Paint Kit (Ultivue-2). Cells were incubated for 2 h at room temperature with 1:100 of Goat-anti-Rabbit (D2) antibody diluted in Antibody Dilution Buffer. Lastly, click chemistry was performed. For click chemistry, we prepared a reaction consisting of HEPES pH 8.2 150 mM, amino guanidine 50 mM (#396494, Sigma), ascorbic acid 100 mM (#A92902, Sigma), CuSO4 1 mM, glucose 2% (#G8270, Sigma), Glox (described in STORM imaging) 1:1000 and Alexa647 azide 10 nM (#A10277, Thermo). The sample was incubated in this reaction for 30 min at RT. Repeated washing was done at every step.

Secondary antibody used was donkey-anti rabbit NHS ester (Jackson ImmunoResearch) custom labeled with AF405/AF647 activator reporter dyes as previously described (58).

### STORM imaging and analysis

Imaging of H2B in single color images with spectrally different dyes was performed on a custom-built inverted microscope based on Nikon Eclipse Ti frame (Nikon Instruments). The excitation module was equipped with five excitation laser lines: 405 nm (100 mW, OBIS Coherent, CA, USA), 488 nm (200 mW, Coherent Sapphire, CA, USA), 561 nm (500 mW MPB Communications, Canada), 647 nm (500 mW MPB Communications, Canada) and 750 nm (500 mW MPB Communications, Canada). Each laser beam power was regulated through AOMs (AA Opto Electonics MT80 A1,5 Vis) and different wavelengths were coupled into an oil immersion 1.49 NA objective (Nikon). An inclined illumination mode (59) was used to obtain the images. The focus was locked through the Perfect Focus System (Nikon). The fluorescence signal was collected by the same objective and imaged onto an EMCCD camera (Andor iXon X3 DU-897, Andor Technologies). Fluorescence emitted signal was spectrally filtered by either a Quad Band beamsplitter (ZT405/488/561/647rpc-UF2, Chroma Technology) with Quad Band emission filter (ZET405/488/561/647m-TRF, Chroma), or a custom Penta Band beamsplitter (ZT405/488/561/647/752rpc-UF2) with a Penta Band Emission filter (ZET405/488/561/647-656/752m) as in (40). STORM and STORM+PAINT raw image data were acquired at 20 ms per frame. 488, 560, 647 or 750 nm lasers were used for exciting the reporter dye and switching it to the dark state, and a 405 nm laser was used for reactivating the reporter dye. An imaging cycle was used in which one frame belonging to the activating pulse laser (405) was alternated with three frames belonging to the reporter dye. Single color imaging was performed using a previously described imaging buffer (58): 100mM Cysteamine MEA (#30070, Sigma-Aldrich), 5% glucose (#G8270, Sigma-Aldrich, 1% Glox (0.5 mg/ml glucose oxidase, 40 mg/ml catalase (#G2133 and #C100, Sigma-Aldrich)) in PBS. A minimum of 45 000 frames were obtained for every image.

For dual color, 3D SMLM images were acquired with a commercial N-STORM microscope (Nikon) equipped with a CFI HP Apochromat TIRF 100 × 1.49 oil objective, an iXon Ultra 897 camera (Andor) and a Dual View system (Photometrics DV2 housing with a T647lpxr dichroic beamsplitter from Chroma). The dual view allowed us to split the image on the full chip of the camera based on emission wavelength. 647 nm laser was used to excite the DNA labeled with AlexaFluor 647 using a power density of ∼3 kW/cm^2^. Simultaneously, in order to perform PAINT, the 560 nm laser was used with ∼0.8 kW/cm^2^ power density to excite the dye attached to the imager strand. The 405 laser was used for reactivating AlexaFluor 647 via dSTORM during acquisition. The 488 laser at ∼0.1 kW/cm^2^ power density was used to illuminate the fiduciary beads, which were used for drift correction and chromatic alignment. The imaging cycle was composed by 19 frames of simultaneous 405, 560 and 647 nm activation interspersed with one frame of 488 nm illumination. The yellow beads imaged with the 488 nm laser were visible in both the red and orange channel, albeit dimly in the red channel. In all cases, Alexa 647 was progressively reactivated with increasing 405 nm laser power during acquisition up to a maximal power density of 0.020 kW/cm^2^. The imaging buffer was composed of 100 mM Cysteamine MEA, 5% glucose, 1% Glox and 0.75 nM Imager strand (I2-560 Ultivue) in Ultivue Imaging Buffer. Localizations were extracted from raw images of bead calibration, STORM and STORM+PAINT data using Insight3 standalone software (kind gift of Bo Huang, UCSF).

The N-STORM cylindrical lens adaptor was used for STORM+PAINT data acquisition to obtain 3D localization data, as previously described (60). Briefly, calibration data was first acquired by imaging subdiffraction limit size beads (100 nm Tetraspeck, ThermoScientific, T7279) in PBS adsorbed to clean glass at low enough dilution to enable single bead visualization. Using the NIS software STORM module, *Z*-calibration data was recorded as the microscope stage was moved in 10 nm steps over a 1.6 μm range and through the objective focal plane to image the elliptically shaped beads as they first elongated vertically, then horizontally. Bead localizations were extracted using the aforementioned Insight3 software through elliptical Gaussian fitting to extract horizontal and vertical width values (*W_x_* and *W_y_*, respectively). These widths were plotted versus the distance of the localization from the imaging focal plane, then fit with a third order polynomial, as previously established (60). The coefficients of the polynomial function were input into the Insight3 software to enable assignment of an axial, *z*, position to elliptical localizations from STORM+PAINT data in addition to their lateral, *x* and *y*, positions. Rendering of DNA and histone images was performed using a summation of uniform Gaussian peaks having a fixed standard deviation of 20 nm.

### STORM-PAINT workflow

STORM localizations of DNA structure in the red 647nm channel were overlaid with PAINT localizations of histone structure in the orange 560 nm channel through a defined, multistep data workflow. Firstly, the same Tetraspeck beads used for *Z*-calibration were utilized to define a single, second order polynomial surface transfer function to overlay the two colors in *x* and *y* when imaged with the dual view. To this end, beads in 10–15 separate fields of view were imaged for 100 frames. The localizations were grouped into bead-clusters and their center identified. The bead centers from all fields of view were combined to create a pseudo-high density image map to define the registration between the orange and red channel and remove residual chromatic aberrations.

Raw STORM+PAINT image data was obtained using the aforementioned microscope hardware, including the dual view and NSTORM cylindrical lens, to record a minimum of 200 000 frames with 20 ms camera exposure. Red-channel STORM localizations were extracted in 3D from the 20 ms images. For the orange channel PAINT localizations, five sequential image frames were first summed together to obtain an effective 100ms camera exposure, then the 3D localizations were extracted from these summed frame images. The five-frame summed images were used also to extract red-channel fiduciary bead localizations, and thereby improve the bead's signal-to-background ratio, while the orange-channel fiduciary bead localizations were extracted from the raw 20 ms images.

All orange-channel localizations were first overlaid upon the red channel localizations in *x* and *y* using the second order polynomial surface transfer function. Next, bead localizations in each color were grouped into bead-clusters and used to extract and correct the drift trajectory for each color throughout data acquisition. Following drift correction of the datasets, the fiduciary bead positions were used to refine the lateral alignment of the two datasets in *x* and *y* using a linear affine transformation. Finally, the axial positions of the fiduciary beads were used to apply a rigid *Z*-translation to the orange dataset and fully overlay the 3D DNA and histone datasets. Once aligned, a 120 nm slice centered near the imaging focal plane (i.e. *Z* = 0) was selected for further co-structural analysis. This workflow was automated using functions developed in MATLAB version 2013a and 2016a.

### Voronoi tesselation analysis

Voronoi tesselation analysis was performed in MATLAB 2016a in a fashion similar to (28). First, the lateral *x*, *y* localizations were input into the ‘delaunayTriangulation’ function, and then used to construct Voronoi polygons using the ‘Voronoidiagram’ function. Areas of the Voronoi polygons were determined from the vertices with the function ‘polyarea’. The local density in each data point was defined as the inverse value of the area of the corresponding Voronoi polygon. Voronoi polygons were visualized using the ‘patch’ function, wherein the look up table was mapped to cover 99% of the polygons; the smallest polygons being yellow (high density, >0.02 nm^−2^), larger polygons set to blue, (low density, <0.001 nm^−2^), and the largest 0.5% of polygons set to black.

**Figure 1.**
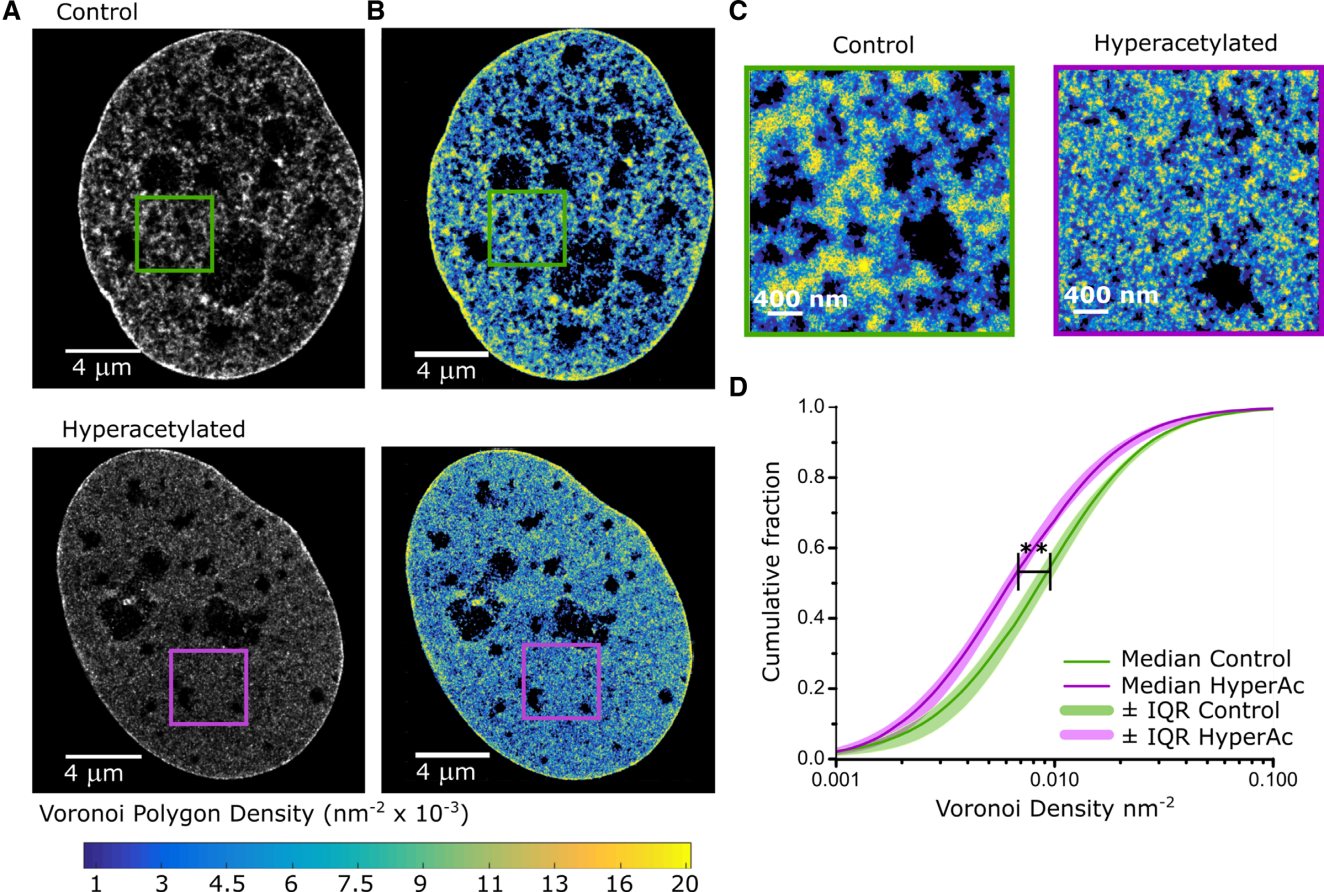
EdC labeling enables super-resolution imaging of DNA structure. (**A**) Cropped nuclear super-resolution images of EdC labeled DNA in control (upper) and TSA-treated (lower) human BJ fibroblast cells rendered using the conventional rendering in which localizations are represented as Gaussians with a fixed width (9 nm). Overlapping Gaussians add up to give rise to higher intensity. (**B**) Super-resolution Voronoi tessellation image of DNA in control (upper) and TSA-treated (lower) fibroblasts quantitatively showing the local variations in DNA density with increased dynamic range. Voronoi polygons are color-coded according to the density (inverse of the polygon area) following the color scale bar (from 0.02 nm^−2^ in yellow to 0.001 nm^−2^ in blue; the largest 0.5% of the polygons are colored black). (**C**) A zoom up of the region within the squares in (B). (**D**) Cumulative distribution of the Voronoi Polygon densities in control (green) (*N* = 6 cells) and TSA-treated (magenta) (*N* = 9 cells) fibroblasts. The light colors show the interquartile range (25–75 percentiles) and the thick, dark lines show the median values; stars indicate statistical significance of the separation between the median of the medians according to Kolmogorov–Smirnoff test with *P* = 0.0022.

3D Voronoi tessellation of Supplementary Figure S2 was performed using the same set of data as in Figure 1. The input in this case was the *x*, *y*, *z* localizations in order to obtain three-dimensional tessels.

### Radial density analysis

Radial density analysis was performed using a custom-written script in Python, version 2.7. The script takes as input the localizations of DNA, as directly obtained by dual-color STORM imaging, and the centroids of the H2B clusters, extracted by a previously developed Matlab script (8). The coordinates of H2B clusters’ centroids were used as center for Voronoi tessellation. Voronoi polygons exceeding the nuclear periphery or in within the nucleoli regions were clipped to a hand-drawn mask (*voronoi_finite_polygons_2d.py function*). The DNA localizations falling within the clipped Voronoi polygon area of a particular clutch center were considered for further analysis. This procedure assigns every DNA localization to a single H2B cluster, preventing the over counting of DNA localizations during the computation. Starting from the H2B clusters’ centroid, disks of increasing radii (steps of 10 nm) were drawn and eventually bounded to the Voronoi polygon (i.e. for circles larger than the polygon, the edges of the polygon were used to clip the disks). The DNA density in within each clipped disk was calculated as the ratio between the number of DNA localizations falling within the clipped disk and the area of the clipped disk (Figure 3B).

### Statistical analysis

Graphpad Prism (v5.04) and Matlab 2016a were used for Statistical analysis. Unpaired two way Anova with Bonferroni multiple comparison test against not treated was used for Cell Cycle experiments.

DNA Voronoi density data in Figure 1D and S2C were obtained by binning the Voronoi density distributions for the six control cells and nine TSA-treated cells into 300 logarithmically spaced bins ranging from 0.1 × 10^−9^ to 0.94 nm^−2^ for Figure 1D and from 5 × 10^−7^ to 0.0025 nm^−3^ for Supplementary Figure S2C. This encompassed the maximal range of the 15 datasets. The median value and interquartile range for each bin was calculated and used to create Figure 1D and S2C; *P*-value for two-sample Kolmogorov–Smirnov test between median value distributions is 0.0022 for both calculations.

The similarity matrix of Figure 4B is calculated in MATLAB 2016a using the ‘kruskalwallis’ function to calculate *P*-value resulting from the Kruskal-Wallis test. This non-parametric version of ANOVA is applicable to the non-Normally distributed data we encounter and calculates the likelihood that rank means from two groups are drawn from the same distribution. To this end, the density of DNA localizations falling within pairs of search discs around H2B clusters is input into the function and the resulting *P*-value matrix comparing all off-diagonal search discs is shown in Figure 4B. This analysis was done by comparing DNA density falling within a given search disk in all clusters of all cells against DNA density in all clusters of all cells falling within a smaller or larger search disk. The diagonal elements were not calculated because the resulting *P*-value would indicate experimental reproducibility rather than provide information regarding changes in DNA density over a distance. Search discs having highly similar DNA densities give rise to larger *P*-values and are color-coded in yellow, whereas discs having low similarity give rise to small *P*-values and are color-coded in blue.

This analysis is used in Supplementary Figure S5A and B. In Supplementary Figure S5A, only a random percentage of the total number of clusters is used for the calculation. In Supplementary Figure S5B, the DNA signal has been rigidly shifted in +*x* and +*y* for the given number of nm, and the radial analysis has been recalculated prior to similarity matrix plotting.

The nearest neighbor distance (NND) analysis of Supplementary Figure S6 was performed by calculating the distance between each H2B cluster obtained centroid to its closest cluster. The centroids of the H2B clusters were obtained using our previously described Matlab script (8).

**Figure 2.**
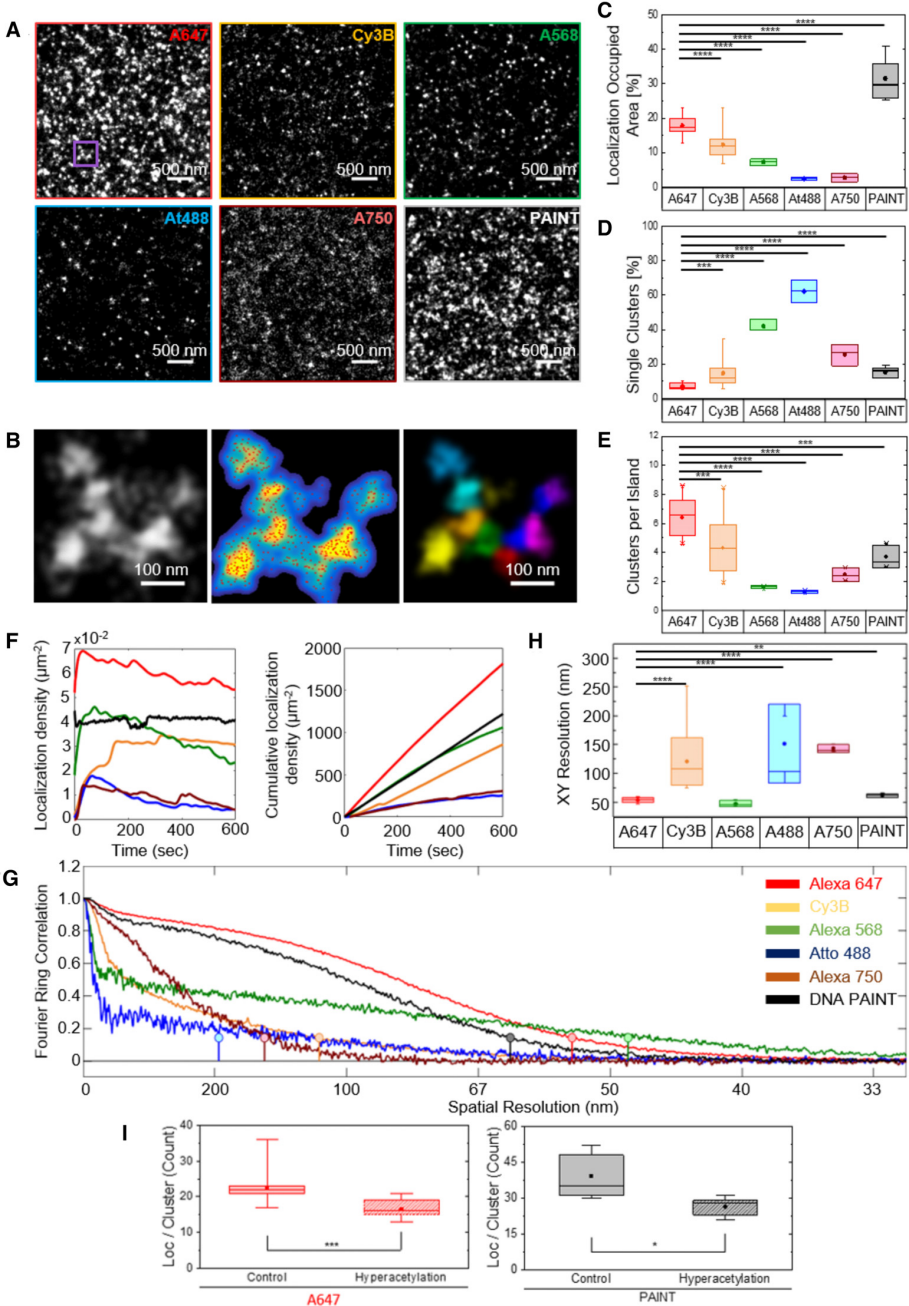
2D super-resolution imaging of H2B with alternative super-resolution compatible dyes. (**A**) Representative 2D super-resolution images of the histone H2B obtained using fluorophores AlexaFluor647, Cy3B, AlexaFluor568, Atto488, AlexaFluor750 or DNA-PAINT super-resolution imaging. PAINT was performed exclusively using a dye excited by 560 nm laser light. (**B**) (left) Zoom up of the region inside the purple square; (middle) Gaussian-based rendering of the same region as a density image from low (cyan) to high (yellow) density and the super-resolution localizations (red); (right) Result of the cluster analysis, each cluster identified using the cluster analysis is color-coded with a different color. In some cases, colors repeat such that distinct clusters may by chance have the same color. We refer to the group of clusters that are in close proximity as ‘islands’ of clusters. (**C**) Percentage of nuclear area occupied by localizations in super-resolution images of H2B recorded using different fluorophores or PAINT super-resolution imaging. The occupancy was calculated by first binning localizations into grids having either a 20 nm super-resolved or a 160 nm diffraction-resolved pixel size. Then the ratio of the summed area occupied with the 20 nm size to the summed area occupied with 160 nm size was calculated and converted to percentage. (**D**) The percentage of isolated, single H2B clusters (i.e. clusters identified to be the only cluster within an ‘island’, see panel B) relative to the total number of H2B clusters identified following cluster analysis of SMLM image data using different fluorophores or PAINT. (**E**) Number of clusters per ‘island’ (panel B) in super-resolution images of H2B recorded using different fluorophores or PAINT. (**F**) (left) Number of localizations per unit area in each frame or (right) cumulative number of localizations per unit area over time during acquisition of raw SMLM image data. H2B was imaged with different fluorophores in STORM or PAINT techniques and the aforementioned localization densities are plotted for a time duration of 600 s. (**G**) Fourier correlation ring (FRC) analysis (35) of the spatial correlations computed from the H2B super-resolution images obtained using different fluorophores in STORM or PAINT SMLM imaging techniques. (**H**) Box plot showing the final image resolution in nm computed from the FRC analysis for each fluorophore or PAINT imaging. The best resolution was obtained for AlexaFluor647, AlexaFluor568 and PAINT. However, AlexaFluor568 gave rise to sparse images (see A, above) in agreement with its FRC plot that shows little long-range structural information in contrast with the AlexaFluro647 and PAINT curves. For box plots in panels C, D, E and H, the box indicates the 25–75th interquartile ranges, the horizontal bar shows the median, the central dot indicates the mean and the whiskers are the min/max values. Note, the *P*-value comparing FRC resolution of AlexaFluor647 to AlexaFluor568 in panel H was 0.063. (**I**) Number of localizations per cluster measured from super-resolution images of H2B recorded using AlexaFluor647 (left) and PAINT super-resolution imaging (right).

The nearest neighbor distance (NND) analysis of Figure 4C was performed by first grouping NND distances between H2B clusters within the same island (see Figure 2) into bins of 20nm spacing. For each bin, the mean cumulative DNA density (i.e. sum of DNA localizations within a search radius divided by the bounded search area) within a search radius of 70 nm from the H2B clutch centroid was calculated for each dataset, four control nuclei and five TSA treated nuclei. Figure 4C was generated by plotting the average of the means across datasets within each bin ± standard deviation.

Statistical significances in panels C, D, E and H of Figure 2 and in Supplementary Figure S6 were calculated using a two-sample unpaired *t*-test. Statistical significance in all panels of Supplementary Figure S3 was calculated using a two-sample Kolmogorov–Smirnov test.

For all tests: ns *P* > 0.05, *P ≤ 0.05, **P ≤ 0.01, ***P ≤ 0.001, ****P ≤ 0.0001.

### Dataset selection & H2B cluster analysis

The inclined illumination utilized on our microscope systems often gave rise to illumination inhomogeneity that was visualized as long, straight areas of a nucleus having sparse localizations. Sub-regions having a more uniform illumination for both 560 and 647 nm laser lines were selected manually and used for H2B and DNA data analysis. A single sub-region was selected for each dataset.

We measured the median lateral localization precision for visualizing H2B *in situ* via DNA-PAINT (36) methodology to be 20.6 nm (16.4, 25.0 interquartile range). The precision was determined by calculating lateral localization precision as the 2D width of a point cloud for localizations visualized in five or more sequential imaging frames (61).

H2B cluster segmentation analysis was performed using the same algorithm previously published (8).

STORM-DNA and PAINT-H2B images were subjected to a quality test as described next and only those images that passed the quality test were accepted for further analysis. DNA datasets were selected according to an estimate for their Nyquist sampling frequency as described previously (22). To this end, two localization densities were calculated for each dataset relative to the nuclear area measured by (i) a diffraction-limited image, and (ii) binning localizations into boxes 20 nm on each side. Dividing the 120 nm slice thickness by this density value, and then taking the cube-root provided an estimate for the sampling frequency within a dataset. The mean ± σ value across all datasets was found to be 30 ± 2 nm/localization. Datasets utilized in DNA analysis (Figure 1C) were required be 32 nm/localization or lower for both densities (i) and (ii).

**Figure 3.**
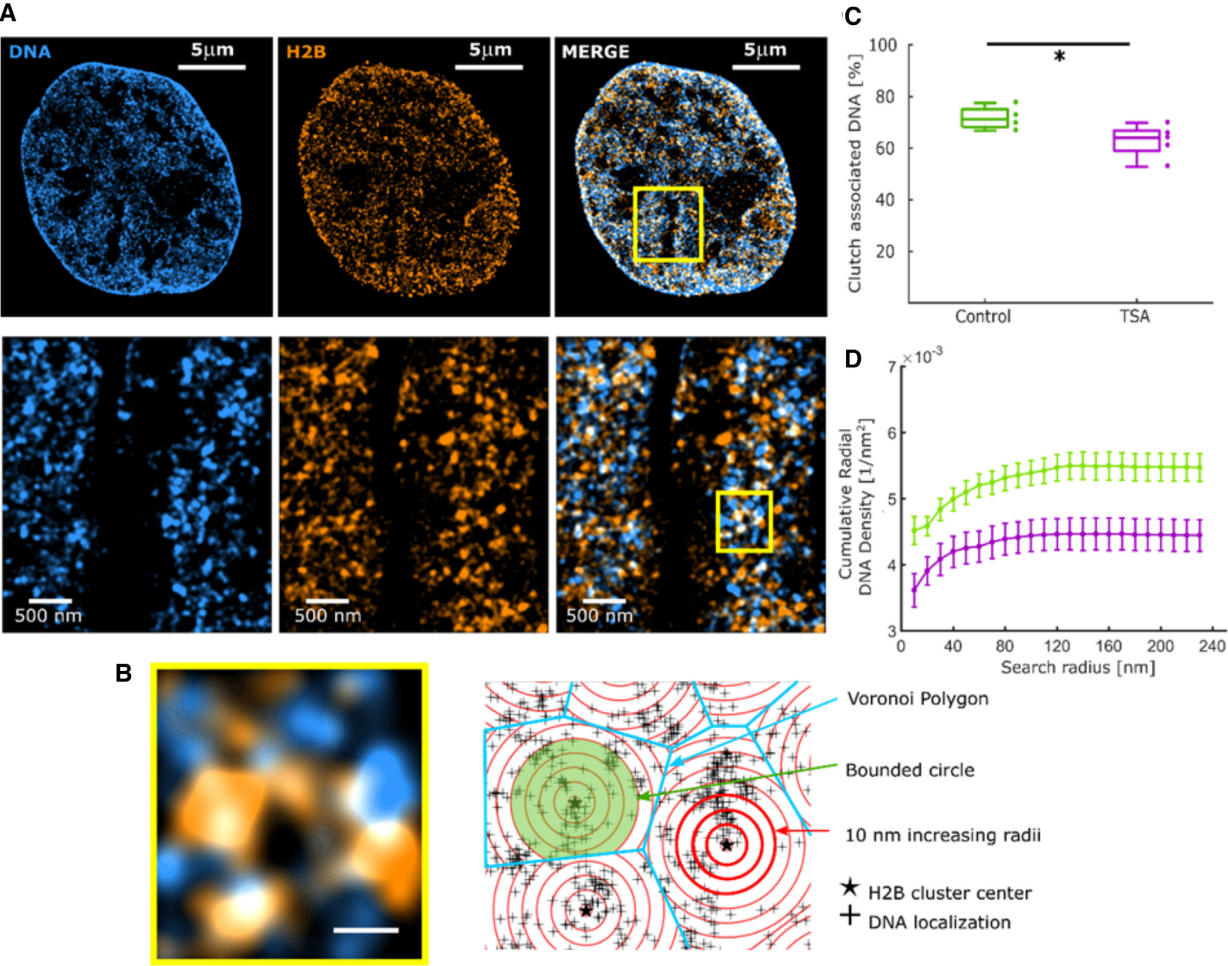
DNA co-localizes with H2B to a lesser extent in TSA-treated cells compared to untreated cells. (**A**) Cropped nuclear super-resolution image in human Fibroblasts of EdC-labeled DNA (cyan) and PAINT image of H2B labeled with anti-H2B antibodies (orange) and the overlay. A zoom of the region inside the yellow box is shown. (**B**) (left) A zoom of the region shown inside the yellow square, (right) scheme of the analysis of clutch-bound DNA. The centers of H2B clusters (stars) are the seeds for the Voronoi polygons (blue) inside which the DNA localizations (black dots) are distributed. Overlaid on top are concentric circles whose radii increase by 10 nm steps. (**C**) Percentage of DNA localizations associated to clutches in wild-type (*N* = 4, green) and TSA-treated (*N* = 5, magenta) fibroblasts for a circle of radius 120 nm bounded by Voronoi polygons (*P*-value 0.0441). (**D**) Cumulative DNA density inside circles of increasing search radii in untreated (green) and TSA-treated (magenta) cells. The dots correspond to the mean, the bars correspond to the standard deviations: the lines connect sequential points and are guides to the eye.

For H2B, thresholds were applied following cluster analysis of an H2B-PAINT dataset using the resulting cluster metrics to determine the quality of a dataset. The three cluster metrics used to select datasets were: (i) localization occupied nuclear area (Figure 2), (ii) clusters per island (Figure 2) and (iii) the nuclear area comprising clusters. This latter metric is a ratio of the area covered by clusters compared to the area covered by localizations, where datasets having low ratios were found to be visually sparser than datasets having a higher ratio. Because the TSA treatment led to H2B reorganization, separate thresholds had to be set for control vs. treated cells. For control Cells, PAINT datasets used for analysis of H2B clusters (Figure 3 and Supplementary Figure S3) were required to have: (i) occupancy > 35%, (ii) clustered area > 15%, (iii) clusters per island > 3; for TSA-treated cells they were required to have: (i) occupancy > 25%, (ii) clustered area > 10%, (iii) clusters per island > 1.5. Dataset selection appeared robust because no datasets were found to have only one or two of the metrics above/below the thresholds. Rather, datasets removed from analysis were found to have all three metrics below the respective thresholds in all cases, while datasets included in the analysis had all three metrics well above the thresholds.

**Figure 4.**
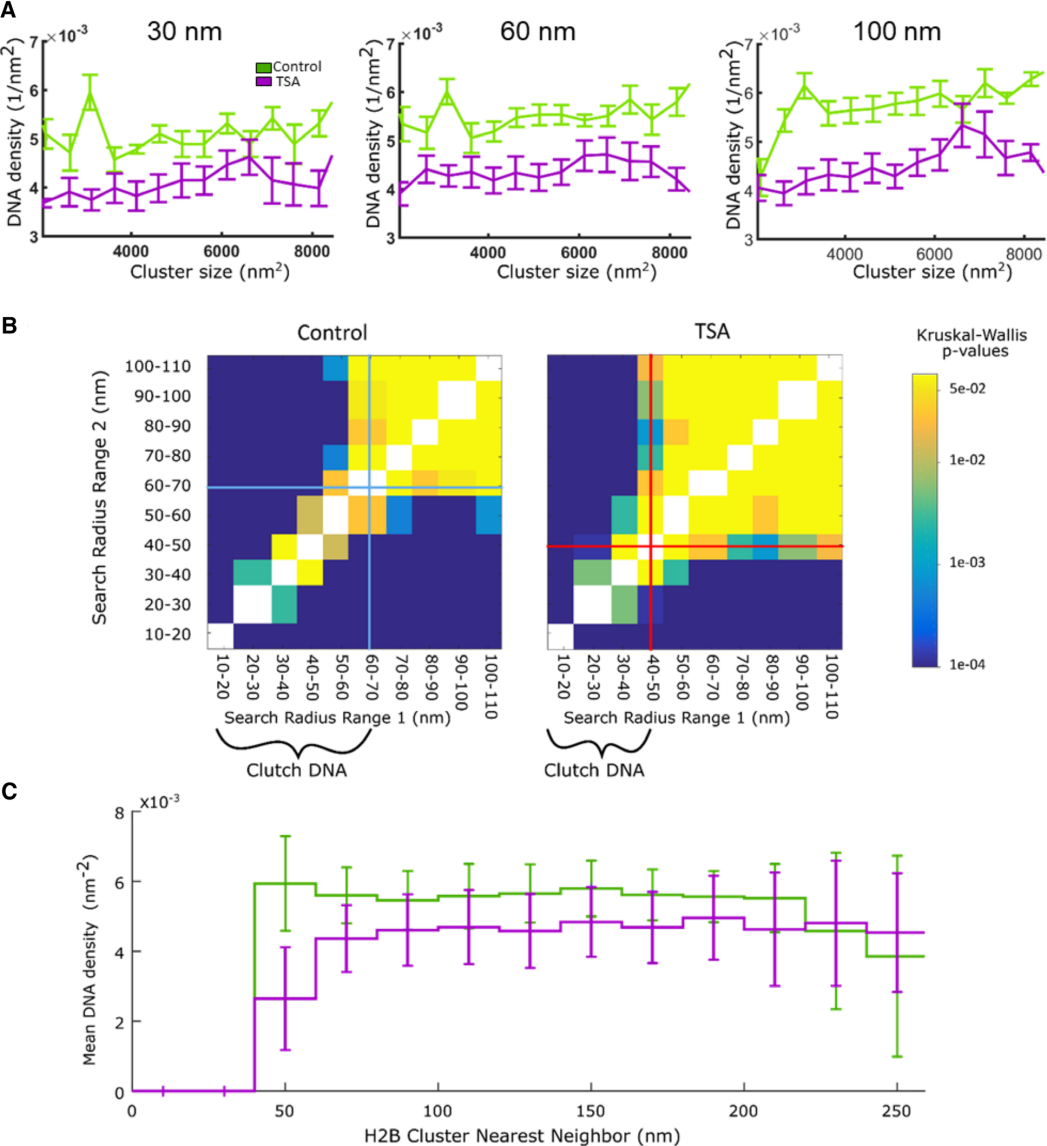
Nucleosome clutch associated DNA undergoes decompaction in TSA-treated cells: (**A**) DNA density in a bound circle of increasing size (30–100 nm) versus cluster (clutch) size measured as area in nm^2^. TSA treated clusters (magenta) have lower density than control clusters (green) independent of cluster size. The line corresponds to the mean of clusters; the bars correspond to the standard deviations. (**B**) Similarity matrix for untreated (left) and TSA-treated (right) cells showing the level of similarity in DNA density within 10 nm discs of increasing radii. The similarity was calculated as a *P*-value from Kruskalwallis test and is shown as a color coding corresponding to the color scale bar (from *P* = 0.0001 in blue to *P* = 0.05 in yellow). The diagonal was set to white and not calculated. Cyan and red lines show the boundary of a switch from low to high similarity in untreated and TSA treated cells, respectively. (**C**) Mean DNA density ± standard deviation as a function of clutch nearest neighbor distance (NND) for untreated (green) and TSA-treated (magenta) cells calculated for a circle of radius 70 nm. The bars show the standard deviation.

Datasets utilized in co-structure analysis of Figure 3, Figure 4 and Supplementary Figure S4 were required to pass the selection criteria for both DNA and H2B.

### Co-localization analysis

To quantify the percentage of DNA localizations associated to nucleosome clutches, a clipped circle of 120 nm radius was drawn and the percentage of DNA localizations inside the disk over the total DNA localization in the polygon was calculated. From this analysis we also quantified the percentage of H2B clusters that have more than 5 DNA localizations inside the clipped disk of 120 nm radius (Figure 3D).

Co-localization analysis of Supplementary Figure S1D was performed using the ‘Coloc2’ plugin in Fiji. Briefly, cell nucleus obtained with both DAPI and EdC-A647 fluorophores were selected as ROIs. The 2D intensity histogram relation between the two signals was obtained and from it, the Pearson's R correlation was calculated.

## RESULTS

### Global DNA compaction decreases in cells with histone tail hyperacetylation

To visualize DNA organization using Stochastic Optical Reconstruction super-resolution microscopy (STORM), human Fibroblast cells (hFb) were labeled with a nucleotide analog 5-ethynyl-2′-deoxycytidine (EdC) (see Materials and Methods) (20,21). EdC was provided at high concentration (5 μM) and for long enough time period (4 days, see Materials and Methods) to ensure dense DNA labeling and enable visualization of global DNA organization. 67 ± 10% of cells were positive for EdC after this treatment (Supplementary Figure S1A) and EdC labeling had minimal impact on cell cycle at the concentration and incubation time used (Supplementary Figure S1B). Subsequent fixation and click chemistry with AlexaFluor647 allowed 3D super-resolution imaging of DNA structure (Figure 1A, upper). Confocal imaging showed that EdC labeling pattern largely overlapped with DAPI labeling, confirming that the EdC uniformly incorporated into all genomic DNA (Supplementary Figure S1C and D). We assessed the localization density in the super-resolution images using a Nyquist sampling (22) criterion and selected those having a sampling frequency of 32 nm/localization or lower for analysis (see Materials and Methods for details). This initial filter ensured that the cells analyzed had a high level of EdC incorporation, high labeling density and hence high spatial resolution of the resulting DNA STORM image. Next, we employed a new quantitative approach based on Voronoi tessellation to analyze DNA spatial organization. Previous methods used for analysis of chromatin structure include statistical analysis methods such as Radial Distribution Function, Ripley's *K* and pair correlation function or segmentation methods such as distance or density based clustering (8,23–27). For example, single molecule based super-resolution imaging of fluorescently tagged H2B was combined with Radial Distribution Function analysis to reveal expression dependent and radiation induced changes to nucleosome organization (27). This early work gave valuable information on chromatin organization, revealing correlations at length scales below 300 nm (26,27), which is consistent with more recent super-resolution studies using Ripley's K and distance based cluster analysis (6,8). However, Ripley's *K* type statistical analysis methods produce only an average estimate of the length scale of spatial correlations in the image without any information on heterogeneity and the local spatial context of the density distribution. Clustering methods, on the other hand, are only suitable for segmenting small nanodomains of roughly uniform size and fail when the image contains a mixture of structures with varying length scales, which is the case for super-resolution images of DNA. Hence, while clustering algorithms work well to segment clustered nucleosome structures, there is no *a priori* reason to limit analysis to only clustered regions of the DNA structure. To circumvent the limitation of these previous methods and analyze DNA density distribution within the nucleus in an unbiased manner, we took advantage of Voronoi tessellation (see Materials and Methods) (28,29). Voronoi tessellation can directly determine the local, precise density in the proximity of each localization in the super-resolution image without imposing a box-size, a search radius or other user-defined parameters (30,31), unlike other methods including Ripley's *K* function. To our knowledge, our work is the first example of Voronoi analysis applied to segment and quantitatively characterize DNA local density distribution and nano-structure within the nucleus.

To this end, super-resolution images of DNA were segmented using Voronoi tessellation (28,29) and color coded according to the inverse of the area of Voronoi polygons (Supplementary Figure S2A, Figure 1B, upper and C, left). The Voronoi polygon area is inversely related to DNA density, with higher density regions corresponding to smaller Voronoi polygons and vice versa (Supplementary Figure S2A). Although localizations were extracted in 3D, the analysis was performed on a 2D projection of a thin, 120 nm slice through the center of the nucleus, which contained approximately 33% of all localizations (Supplementary Figure S2B). The 120 nm z-thickness was selected so as to include a sufficiently high density of localizations for structural analysis while minimizing 2D projection effects that can arise when imaging over the 600–800 nm range accessible in the STORM technique. This volume is also in a similar range as recent electron tomographic reconstruction of DNA organization (9,11). Compact, heterochromatic regions typically located at the nuclear edge and surrounding the nucleoli in human fibroblasts were characterized by high DNA density in the super-resolution images (Figure 1B, upper), suggesting that DNA density is indeed a quantitative measure of chromatin compaction and presence of heterochromatin. Consistent with previous work that measured a 5- to 7-fold increase in heterochromatin compaction compared to euchromatin using Hoescht labeling and conventional fluorescence imaging (32), our results also showed that the Voronoi density of heterochromatic regions (defined as the smallest 15th percentile of the Voronoi polygon areas) was 6.93-fold higher compared to that of euchromatic regions (defined as the largest 85th percentile of the Voronoi polygon areas). Treatment with the Histone Deacetylase (HDAC) inhibitor Tricostatin A (TSA) has been shown to lead to hyperacetylation of histone tails, chromatin opening and increased transcriptional activity (33). We therefore treated cells with TSA during the final 24 h of EdC incubation and labeled them with click chemistry, as before, to quantitatively compare global DNA density of control and hyperacetylated cells. The EdC labeling efficiency, measured as the number of fluorophore localizations detected per unit cell area, was unchanged between control and TSA treated cells (Supplementary Figure S1E). Voronoi tessellation on the 120 nm 2D sections demonstrated that TSA treatment induced a decrease in the median DNA density (Figure 1D) consistent with genome wide decompaction of DNA induced by hyperacetylation. 3D Voronoi tessellation analysis of the entire DNA images showed the same results (Supplementary Figure S2C), suggesting that our conclusions are not affected by the 2D projections used in our analysis.

### Combined PAINT and STORM super-resolution microscopy allows visualization of histone and DNA co-organization at the nanoscale level

Previously, we showed that nucleosomes are organized in heterogeneous groups, which we termed nucleosome clutches, and demonstrated that nucleosome clutch size decreased in TSA-treated human fibroblasts (8). To gain further quantitative insight into how the DNA is compacted by multiple nucleosomes present within an individual clutch, we aimed to carry out two-color super-resolution imaging of DNA and histone H2B to simultaneously visualize remodeling of nucleosome clutches together with their associated DNA. Since the click chemistry used to label DNA offers a limited choice for reliable super-resolution compatible, photoswitchable fluorophores (mainly AlexaFluor647), we aimed to identify a second fluorophore with a different emission wavelength to carry out the immunofluorescence labeling of histones. To this end, we first tested a wide range of fluorophores previously reported to be compatible with super-resolution microscopy (34) and compared the histone clutch structure to those obtained by the best-performing AlexaFluor647 (Figure 2A). The histone data were analyzed using our previously developed distance-based cluster identification algorithm and the localizations were segmented into nucleosome clutches (8) (Figure 2B).

Detailed analysis of these data showed that most fluorophores gave rise to sparse appearance of the clutches in the rendered images compared to those obtained by AlexaFluor647 (Figure 2A and C). Indeed, the nuclear area occupied by fluorophore localizations was significantly lower for Cy3B, AlexaFluor568, Atto488 and AlexaFluor750 compared to AlexaFluor647 (Figure 2C). Nucleosome clutches in STORM images of AlexaFluor647 typically cluster together, with multiple clutches in close spatial proximity of one another (Figure 2B), likely corresponding to the higher order folding of the chromatin fiber. We refer to these nucleosome-clutch-rich regions as clutch ‘islands’. The presence of these clutch islands is consistent with recent super-resolution imaging of single chromosomes, showing multiple DNA nanoclusters in close proximity (25). However, nucleosome clutches imaged with alternative fluorophores were mostly isolated in space, had significantly fewer neighboring clutches and hence did not form clutch islands (Figure 2D, E). We attribute these effects to the poor photoswitching properties of the alternative dyes, in particular in the nuclear environment. Indeed, the localization density per frame was substantially higher for AlexaFluor647, giving rise to a much higher cumulative number of localizations in the same image acquisition time compared to other fluorophores (Figure 2F). Accordingly, the final image resolution computed using the Fourier Ring Correlation (FRC) analysis (35) (Figure 2G) was significantly improved for super-resolution images of histones acquired using AlexaFluor647 compared to other fluorophores (Figure 2H).

To circumvent this problem, we next combined two different localization-based super-resolution imaging modalities: STORM to image DNA and PAINT to image histones. The DNA-PAINT (36), referred here simply as PAINT for clarity, does not rely on the use of imaging buffers and fluorophore photoswitching. Instead, the on-off blinking of fluorophores is achieved by the transient, reversible binding of fluorophore labeled ‘imager oligo strands’ to the complementary ‘docking oligo strands’. The docking strands are conjugated to a secondary antibody (36), which is used for immunostaining and thereby label a target protein. Hence, a wide range of fluorophores can be used for PAINT imaging. Our results showed that PAINT gave rendered super-resolution images of H2B that were both qualitatively and quantitatively comparable to those obtained by STORM imaging using AlexaFluor647, demonstrating the equivalency of the two imaging modalities (Figure 2A, C–H). Importantly, we verified the reliability of the histone nano-structure obtained via PAINT by reproducing our previous biological result obtained using Alexa647-STORM, in which the number of localizations per clutch decreased after TSA treatment (Figure 2I). To combine the two super-resolution modalities for multi-color imaging, we designed an experimental workflow that accounts for the differences in acquisition time needed for STORM and PAINT (see Materials and Methods). Finally, to correct for drift and to align the two images in 3D, we used fiduciary markers that were internalized into cells prior to fixation (Supplementary Figure S3A). To estimate the residual registration error associated with imaging chromatin, we imaged H2B via PAINT in two colors simultaneously (560 and 647 nm laser excitation) to obtain two-color images of the same histone structure (Supplementary Figure S3B). Nearest neighbor distances between H2B clutches present in both colors allowed calculation of a 3D registration error of 31 ± 15 nm (Supplementary Figure S3C). We note that the error measured in this way includes not only the contribution from the registration error but also additional errors. For example, since we used polyclonal primary and secondary antibodies, there are potentially multiple primary and secondary antibodies present on the histone proteins within a given nucleosome (there are two H2B proteins per nucleosome and each can be labeled with more than one primary/secondary antibody complexes). If the two fluorophores bind to different H2B proteins within the nucleosome or different primary/secondary antibody complexes within the nucleosome, the two colors will be spatially segregated due to the bulky size of the antibodies. In addition, the two fluorophores may bind to histones within nearby clutches rather than the histones within the same clutch and the measured NND will then reflect the distance between nearby clutches rather than the registration error. These two contributions combined together bias the registration error measurement towards larger numbers. Indeed, the residual error measured between the position of the fiduciary beads after alignment was much smaller (4.9 ± 4.9 nm). Two-color super-resolution images of histones and DNA imaged using this workflow in control and TSA-treated cells revealed, as expected, a similar pattern of heterogeneous labeling, showing regions enriched in and depleted of chromatin within the nucleus (Figure 3A, Supplementary Figure S4A and B) further demonstrating that both imaging modalities and labels faithfully represent chromatin structure.

### Histone hyperacetylation leads to DNA decompaction through multiple mechanisms

To further quantify the spatial relationship between histones and DNA, we first segmented the nucleosome clutches using our distance based clustering algorithm (8) and determined their center position. We then once again took advantage of Voronoi analysis by using the center position of each clutch as a seed for the tessellation process. This analysis divided the nuclear space into polygons, each polygon surrounding and belonging to one specific clutch (Figure 3B, Supplementary Figure S4B). We next used a circle with a search radius of 120 nm bounded by the Voronoi polygon edges (i.e. if the search radius was larger than the polygon, then the edges of the polygon were used as the boundary) (Figure 3B, Supplementary Figure S4B) and categorized the DNA localizations falling within the bounded circle as ‘clutch-associated’ and those falling outside as ‘clutch-free’ DNA. The 120 nm radius corresponded to about 2.5 times the average standard deviation of the localizations belonging to the H2B clutches, thereby encompassing ∼98% of the nucleosome clutch Gaussian-area. This co-localization analysis, once again, preserves the spatial context and quantitative information about each clutch while defining its area of ‘influence’ in an unbiased way. We found a higher proportion of DNA was nucleosome clutch-associated in control Fibroblasts compared to TSA-treated Fibroblasts (71.7 ± 2.3% in WT Fb and 62.7 ± 2.9% in TSA treated Fb, *n* = 4 and *n* = 5 cells, respectively, (Figure 3C) and this trend was consistent and independent of the search radius selected (Supplementary Figure S4C). These observations are consistent with the results of our previous polymer based modeling of DNA occupancy from single color images of nucleosome clutches, which predicted a decrease in percentage of DNA occupied by nucleosomes after TSA treatment (8). On the other hand, a large percentage of clutches co-localized with DNA in both conditions, 99.9% in control and 99.5% in TSA-treated cells (*P*-value 0.0168) (see Materials and Methods). This result suggests that most histones within clutches are chromatin associated as expected.

To extract a detailed understanding of how the nucleosome clutches compact DNA associated to them, we next determined the DNA localization density within bounded circles having a range of search radii from the clutch center. Nucleosome clutches in TSA treated cells had a lower DNA density around them compared to control cells over the full range of search radii utilized, indicating decompaction of clutch associated DNA after hyperacetylation (Figure 3D). Since clutches in TSA treated cells are smaller than those in control cells (Figure 3A and B, Supplementary Figure S4A and B and Figure 2I), we questioned whether DNA packs more loosely around small clutches in general. To this end, we quantified DNA density as a function of clutch size in both control and TSA treated cells for bounded disks with a search radius ranging between 30 and 100 nm (Figure 4A). This analysis revealed no correlation between clutch size and DNA packing density for both treated and control cells. Therefore, clutches contain an amount of DNA proportional to the number of nucleosomes they contain, and hence the packing density of the total DNA associated with nucleosome clutches is independent of the number of nucleosomes present within them. Interestingly, the DNA density of clutches at all sizes was lower in TSA treated cells compared to untreated cells (Figures 3D and 4A). Therefore, it is the acetylation state of the histones and not the number of nucleosomes within the clutch that determines how tightly clutch-associated DNA is compacted. Acetylation leads to a looser packing of DNA within clutches.

To determine the spatial scale where a nucleosome clutch stops influencing the compaction of DNA surrounding it, we set out to quantify the change in local DNA density around the center of nucleosome clutches. We hence determined a ‘similarity matrix’ by comparing DNA density at increasing distances from the clutch center (Figure 4B). To this end, we first determined the DNA density within 10 nm thick disks of increasing radii (Figure 3B). The disk radius was increased iteratively by 10 nm intervals while keeping the thickness constant, hence moving the ring away from the clutch center stepwise (Figure 3B). We calculated the DNA density within each 10 nm ring from the DNA localizations falling within the ring area and compared it among rings of increasing radii. Similar to pair-correlation analysis, in which correlation drops to random at large distances, we expect high similarity in DNA density at large radii from the clutch center. Our intention was thus to analyze the transition point from low to high similarity at short distances and compare this range between control and treated cells to determine any differences in the scale of DNA packing under these two conditions.

In untreated cells, there was low similarity in DNA densities of disks with radii ranging from 10 to 70 nm (Figure 4B, left, cyan bars), suggestive of variation in DNA compaction over this length scale. Search radii >70 nm, on the other hand, showed high similarity in their DNA density. Therefore, within a 70 nm distance from the clutch center there is large heterogeneity in DNA compaction whereas at larger distances, DNA compaction is unchanged. These results suggest that nucleosome clutches compact DNA within a 70 nm radius from their center in untreated cells. We call the DNA that falls within the low similarity (high variability) region of the matrix ‘clutch’ DNA: i.e. DNA associated to a clutch, which includes both the DNA wrapped around individual nucleosomes as well as the linker DNA in between neighboring nucleosomes. Interestingly, in TSA-treated cells the clutch DNA radius decreased to ∼40 nm (Figure 4B, right, red bars, also compare Figure 3A and B and Supplementary Figure S4A and B). While the spatial scale of the difference between control and TSA-treated clutch DNA is small (∼30 nm) and comparable to our localization precision, this difference is meaningful as our analysis averages over tens of thousands of nucleosome clutches. Such spatial averaging, as also previously demonstrated (37), improves the effective resolution at which we can detect differences in the spatial length scale of clutch DNA in control and TSA-treated cells. Indeed, repeating this analysis with subsets of data points from individual nucleosome clutches clearly demonstrated that the spatial scales of clutch DNA in treated and untreated cells become apparent only when sufficiently large number of clutches are used in the analysis (Supplementary Figure S5A). Further, to rule out any impact on the analysis due to the spatial shift between the histone and DNA images resulting from the finite registration error, we carried out the analysis by progressively shifting the two images with respect to each other in *x* and *y* by increments of 20 nm (Supplementary Figure S5B). The heterogeneity in clutch associated DNA density observed at short length scales progressively decayed and the differences observed in clutch DNA of TSA treated and control cells progressively disappeared as the shift became larger. Therefore, we conclude that the results are robust to small errors in the registration between the two images and become randomized as the proper colocalization between DNA and histones is lost.

Our combined results suggest that the opening of DNA upon histone hyperacetylation is due to decreased nucleosomal occupancy, as revealed by reduced co-localization between DNA and histones as well as more loose packing of clutch DNA. These results are consistent with previous *in vitro* results, which demonstrated a disruption of histone-DNA interactions in nucleosomes having acetylated tails.

### Clutches in spatial proximity influence each other's DNA packing

**Figure 5.**
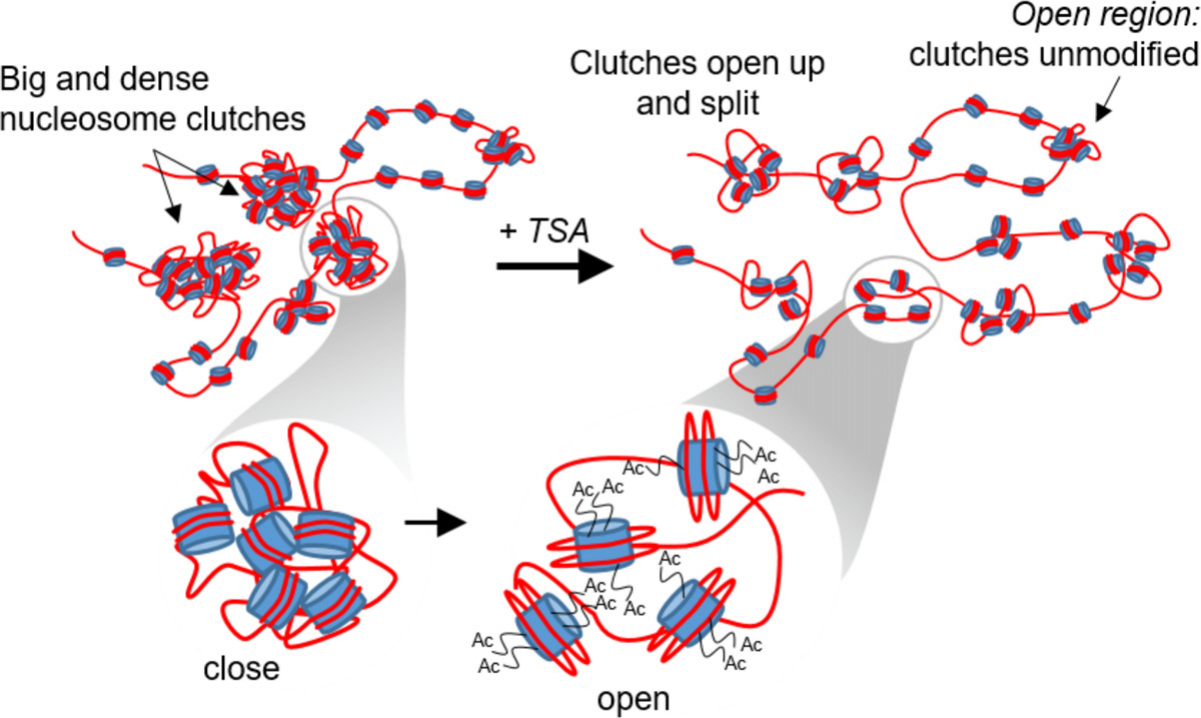
Acetylation changes clutch size and the packing density of clutch DNA. Cartoon model of nucleosomal DNA decompaction upon hyperacetylation. Nucleosome clutches become smaller (see Figure 2I, Figure 3A, B and Supplementary Figure S4A, B), likely due to a combination of large clutches opening and splitting and nucleosome loss. Clutches also compact ‘clutch’ DNA to a lesser extend in hyperacetylated cells (see Figure 3D and Figure 4B). These changes are particularly prominent in highly folded regions of the chromatin containing multiple nucleosome clutches in close proximity (see Figure 4C), suggesting that clutches may influence DNA compaction of neighboring clutches.

In human fibroblasts, clutches are clustered in close spatial proximity of one another, forming ‘islands’ containing several clutches organized into higher order structures, which were hundreds of nanometers in size (Figure 2B). We reasoned that these regions might correspond to the higher order folding of the chromatin fiber, bringing multiple clutches into close proximity to further compact DNA. We next wondered how the DNA packing changes upon hyperacetylation in relation to these folded regions of the chromatin. We compared the DNA density falling within a radius of 70 nm from the clutch center (the clutch DNA for control cells) as a function of the nearest neighbor distances (NNDs) between clutches (Figure 4C). Surprisingly, the largest difference in DNA density between control and TSA treated cells was observed for clutches having an NND of ∼50 nm or smaller (Figure 4C). These results suggest that the largest decompaction due to hyperacetylation happens in folded chromatin regions containing high-order structures, where decompacted DNA from one nucleosome clutch may influence the DNA packing of a nearby, neighboring clutch (Figure 5), thus changing the DNA density of multiple clutches. This decompaction might be due to the presence of a high density of nucleosome tails that become hyperacetylated in these regions, thus generating a large amount of repulsive charges that more strongly disrupt DNA-histone interactions leading to higher DNA decompaction. On the other hand, the more unfolded regions corresponding to more isolated clutches do not undergo major modifications, likely since chromatin in these regions is already in an open state (Figure 5). The inter-clutch NND was smaller in TSA treated cells compared to control cells (Supplementary Figure S6) but the small difference (∼5 nm) is not sufficient to explain the differences observed in DNA density for nearby clutches.

## DISCUSSION

Visualization of DNA fibers at nanoscale resolution within an intact nucleus can potentially reveal details about how chromatin organization relates to gene activation, information that biochemical or genetic approaches can only indirectly infer (38). Super-resolution microscopy has recently revealed the spatial organization of chromatin and transcriptional machinery in mammalian and bacterial cells at length scales of individual genes that were previously inaccessible to light microscopy (8,24,39–43). Labeling of specific genomic regions using Oligopaint technology and imaging with super-resolution (OligoSTORM) uncovered the higher order packing of genomic regions having different epigenetic states (39–41,44,45). By using super-resolution microscopy, we previously revealed the organization of the chromatin fibers by visualizing nucleosome clutches (8). More recently, pulse-chase EdU labeling and click chemistry followed by super-resolution microscopy was used to label the DNA of individual chromosomes (25). These images showed that DNA formed nanodomains, which were further clustered in close spatial proximity, an organization similar to nucleosome clutches that also cluster in close proximity forming ‘islands’. In addition, multi-color imaging of DNA and histone modifications revealed that active histone marks formed small, disperse nanodomains whereas silencing marks formed large clusters that pack DNA more tightly (19). These semi-quantitative and correlative observations suggested that the size of the nucleosome domains may be a determinant of the packing density of DNA and hence the activation/silencing of genes. However, thus far, there had been no quantitative structural analysis probing how DNA packs around multiple nucleosomes within a nanodomain (i.e. clutch), and directly measuring the independent contributions of the nanodomain size and epigenetic state of nucleosomes within a nanodomain to DNA packing.

Here, we carried out multi-color super-resolution imaging of histones together with DNA and highly quantitative spatial analysis to describe (i) how the DNA is compacted by the nucleosome clutches and (ii) how this compaction changes after histone acetylation. Our results showed that DNA becomes less compact upon TSA treatment, which is consistent with previous biophysical studies that reported increased accessibility of chromatin to high molecular weight dextrans after TSA treatment (46). In addition, fluorescence recovery after photobleaching (FRAP) showed increased histone H2A mobility after TSA treatment (47). This previous work, taken together with our results, suggests that histones may be more dynamically exchanged from de-condensed chromatin.

We further showed that the percentage of DNA co-localizing with nucleosomes decreased after TSA treatment. Previously, we modeled chromatin organization using a simple polymer model in which nucleosome occupancy was varied (8). This model predicted that the experimentally observed decrease in nucleosome clutch size after TSA treatment could be recapitulated through a nucleosome removal mechanism leading to lower nucleosome occupancy. Consistent with this synthetic model, our two-color imaging data showed a decrease in the percentage of DNA co-localizing with nucleosome clutches. Disruption of both histone-DNA and inter-nucleosome interactions (16,17) may lead to destabilization, sliding and eviction of nucleosomes, resulting in a lower DNA occupancy. Alternatively, disruption of inter-nucleosome interactions (18) may lead to splitting apart of large nucleosome clutches, leading to opening up and release of previously undetectable nucleosome-free linker DNA.

Further, in the super-resolution image data, we discriminate DNA associated to nucleosome clutches and term it ‘clutch’ DNA. Our results demonstrate that the density and length-scale of clutch DNA, and hence its level of compaction, is dependent on histone tail acetylation but not on the size of the clutch. Hence, our results are in line with recent multi-color super-resolution imaging of DNA and histone modifications, which showed a correlation between abundance of H3K9ac histone with regions having more sparse DNA localizations (19). However, our results go further to show that the decreased DNA density in these regions is not due to the smaller size of the domains containing H3K9ac histones but rather to the epigenetic state of these domains.

We found DNA decompaction due to acetylation to be most prominent in highly folded regions of the chromatin, where clutches influence the DNA compaction of other neighboring clutches in close vicinity. It is likely that many histone tails of groups of clutches within large domains undergo hyperacetylation and hence the DNA compaction is perturbed to the highest level in these regions. Ultimately, this acetylation-dependent DNA decompaction regulates accessibility of RNA polymerase II and transcription factors to DNA regulatory sequences that may lie in these highly folded and compacted regions of the chromatin. Indeed, in fibroblasts we previously showed that RNA polymerase II is associated to the small and less dense clutches, similar to the ones generated after TSA treatment (8). A gene likely contains multiple clutches and it would be interesting in the future to investigate if RNA polymerase II associates mostly with the decompacted DNA generated between the neighboring nucleosome clutches and within islands encapsulating coding genes. Such visualization could ultimately be informative to study transcriptional activation. We and others (25,48–51) have developed methods to image specific genomic sites. In combination with the highly quantitative analysis methods we developed here, including Voronoi based density and similarity matrix analysis of DNA compaction, these new tools pave the way to study DNA packing at clutches located in specific regulatory regions with nanometric resolution, in order to correlate function with the structure of DNA.

Finally, future developments in single molecule imaging and super-resolution microscopy with improved spatial resolution can potentially enable the visualization of chromatin structure at the level of individual nucleosomes in intact nuclei. Single molecule fluorescence resonance energy transfer (FRET) has higher spatial resolution (1–10 nm) than super-resolution microscopy and has been extensively used to probe DNA-nucleosome interactions and nucleosome remodeling *in vitro* (52–55). However, the crowded cell environment makes it challenging to translate these studies *in vivo*. Recently, spatial resolution below 10 nm was achieved in PAINT imaging by using modified aptamers to label cellular proteins with small tags (56). Development of new aptamers suitable for labeling endogenous histone proteins will potentially make it possible to visualize individual nucleosomes at high resolution in cells. In addition, new methods, such as Oligopaint-derived approaches, have been developed to visualize specific genomic regions with ultra-high spatial resolution. These methodologies include Optical Reconstruction of Chromatin Architecture (ORCA), in which short sections of genomic regions (few kb) are tiled with oligo probes (57). These oligo probes are imaged and localized sequentially providing spatial resolution reconstruction of gene architecture with nanometer scale. A combination of these approaches with the ones present in this paper can in the future potentially enable visualization of chromatin structure with nucleosome level spatial resolution.

## Supplementary Material

gkz593_Supplemental_File

## Notes

Present address: Jason Otterstrom, IDEA Bio-Medical, 08860 Castelldefels, Spain.
