# Supplementary material for: Super-resolution microscopy reveals how histone tail acetylation affects DNA compaction within nucleosomes in vivo
Source: Nucleic Acids Res. 2019 Jul 9;47(16):8470–84. doi: 10.1093/nar/gkz593 (PMC6895258; doi:10.1093/nar/gkz593)
Supplement: gkz593_Supplemental_File [file gkz593_supplemental_file.pdf]

Supplementary Figures

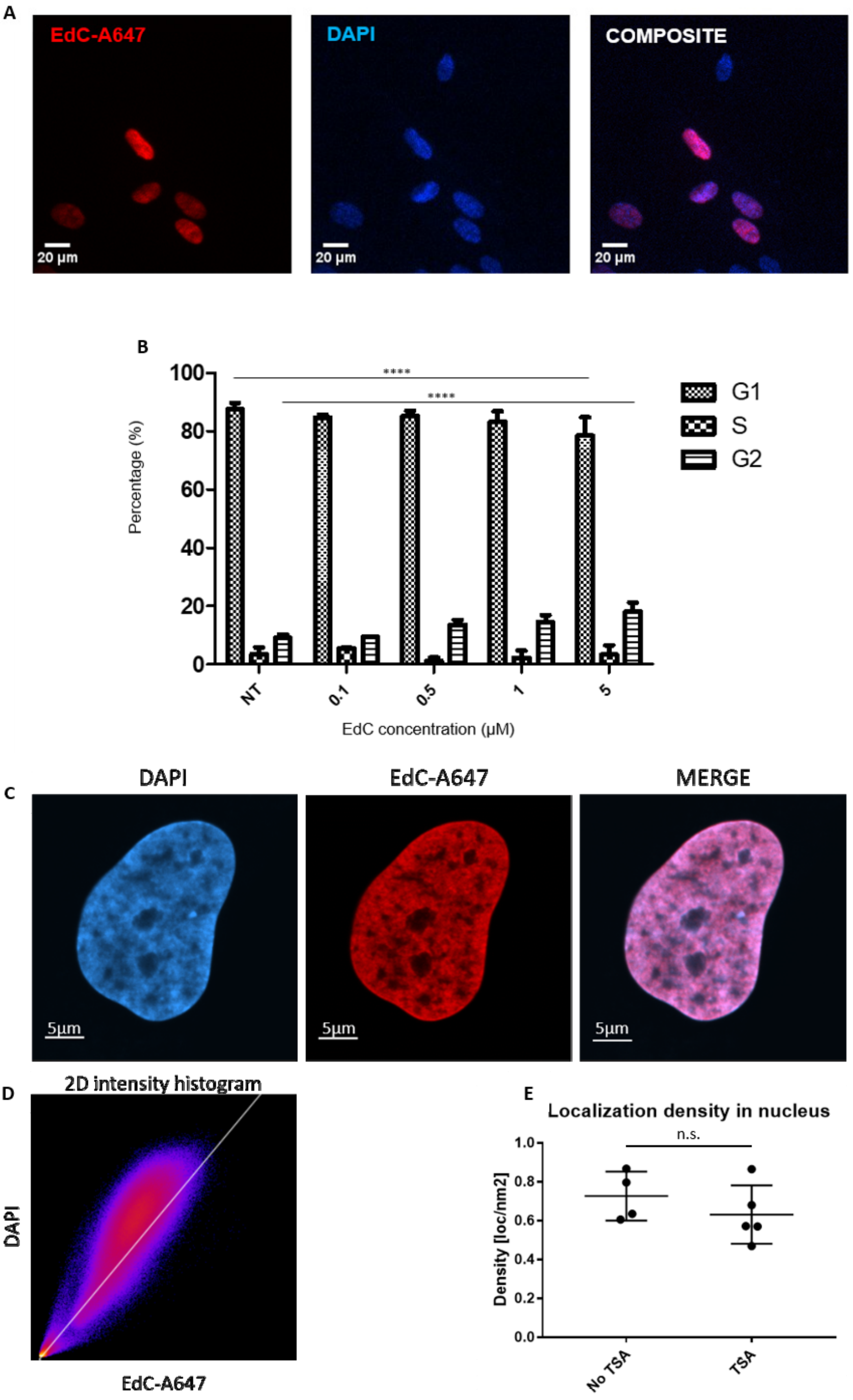

Figure S1. **Effect of EdC labeling on cells. (A)** Representative wide-field image of EdC treated click-labelled cells. (left) positive EdC-A647 labelled cells; (center) nuclear DAPI staining; (right) composite. **(B).** Percentage of cells in G1, S and G2 phase of the cell cycle after labeling with different concentrations of EdC. NT refers to Not Treated. Cells were sorted by flow cytometry after staining the DNA using Propidium Iodide. The quantification was done using the commercial program ModFit LT (Allen 1990), for unbiased quantification of cell cycle phase populations. 87.5% of cells were in G1 in the absence of labeling (NT) and 78.4% of cells were in G1 after labeling with 5 µM of EdC. Stars indicate statistical significance according to an unpaired two way Anova with Bonferroni multiple comparison test against not treated $p<0.0001$. N=3 biological replicates and N=2-3 technical replicates per experiment. **(C)** Representative confocal microscopy image of nuclear labeling with DAPI **(left)** versus nuclear labeling with EdC followed by click chemistry **(center)** in the same cell and the overlay of the two labels **(right). (D)** 2D intensity histogram of EdC-A647 signal versus DAPI signal. Color code represents frequency of intensities. Pearson's R Value:0.98 **(E)** Localization density in nucleus measured as total number of localizations in the analyzed nuclear regions per total area of the region. P-value>0.3 according to unpaired t-test.

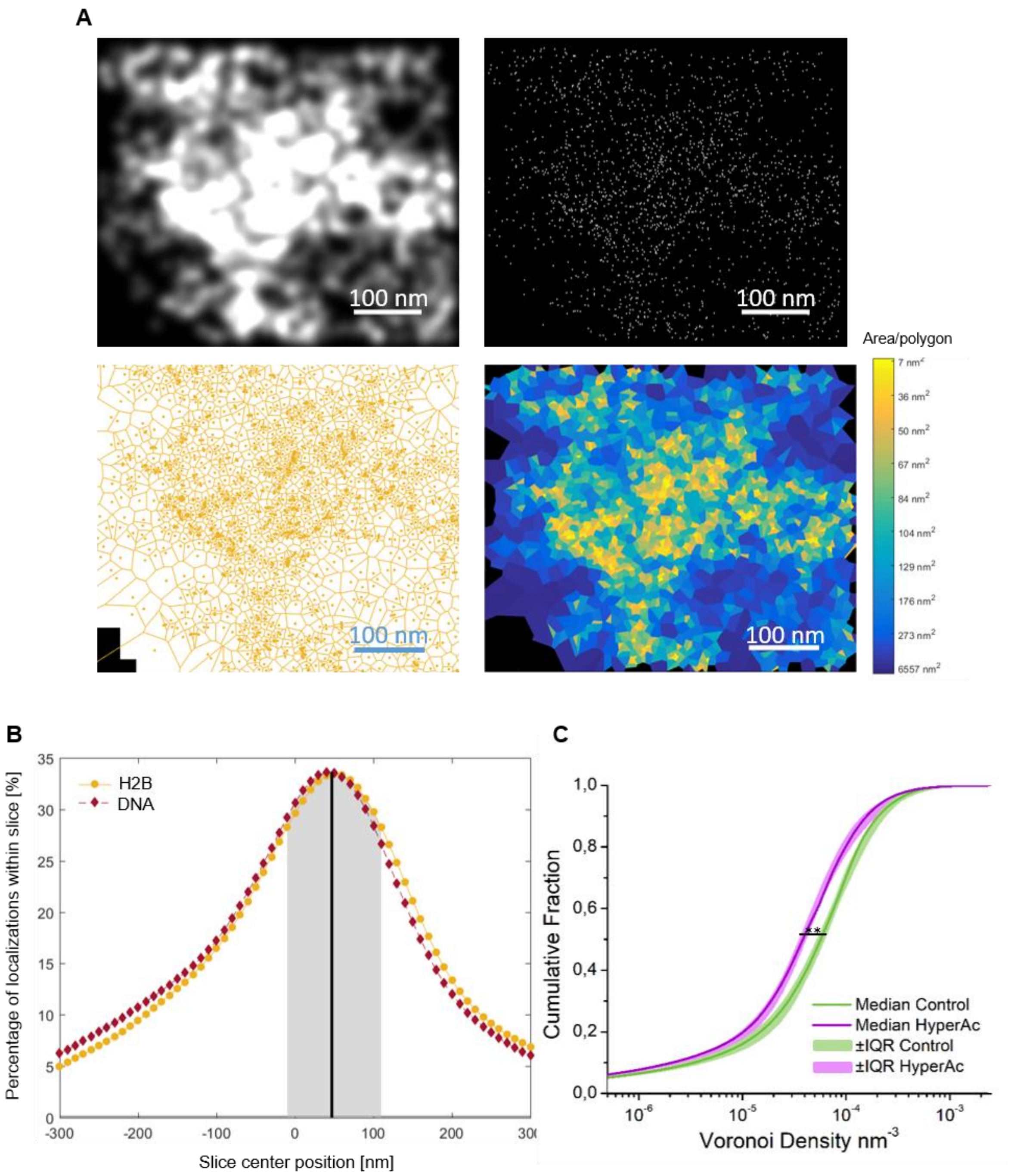

Figure S2. **3D data selection and Voronoi analysis.** **(A)** Voronoi data analysis schematic. Top left: Conventional rendering of DNA data. Every localization is rendered as a fixed-size 2D-Gaussian. The intensity scales with the number of overlapping Gaussians. When many Gaussian overlap, the image saturates, concealing the features of higher density structures. Top right: Raw localization data. Every localization is rendered as a point. Bottom left: voronoi tessellation of DNA localization data. Every localization is used as the seed for a tessel. Bottom right: Voronoi density rendering based on the tessel size, providing improved dynamic range over the conventional rendering and quantitative discrimination of local density variation**.** **(B)** Percentage of localizations as a function of the z-position from the central focal plane (at 0) for two-color datasets following axial registration. The orange and red dots correspond to the two color-channels for H2B and DNA, respectively. The light grey area under the distribution indicates the 120nm thick z-slice used for the analysis, which encompassed ~33

% of localizations for both color channels across all datasets. **(C)** Cumulative distribution of the 3D Voronoi Polygon densities in control (green) (N=6 cells) and TSA-treated (magenta) (N=9 cells) fibroblasts. The light colors show the interquartile range (25-75 percentiles) and the thick, dark lines show the median values; stars indicate statistical significance of the separation between the median of the medians according to Kolmogorov Smirnoff test with $p = 0.0022$

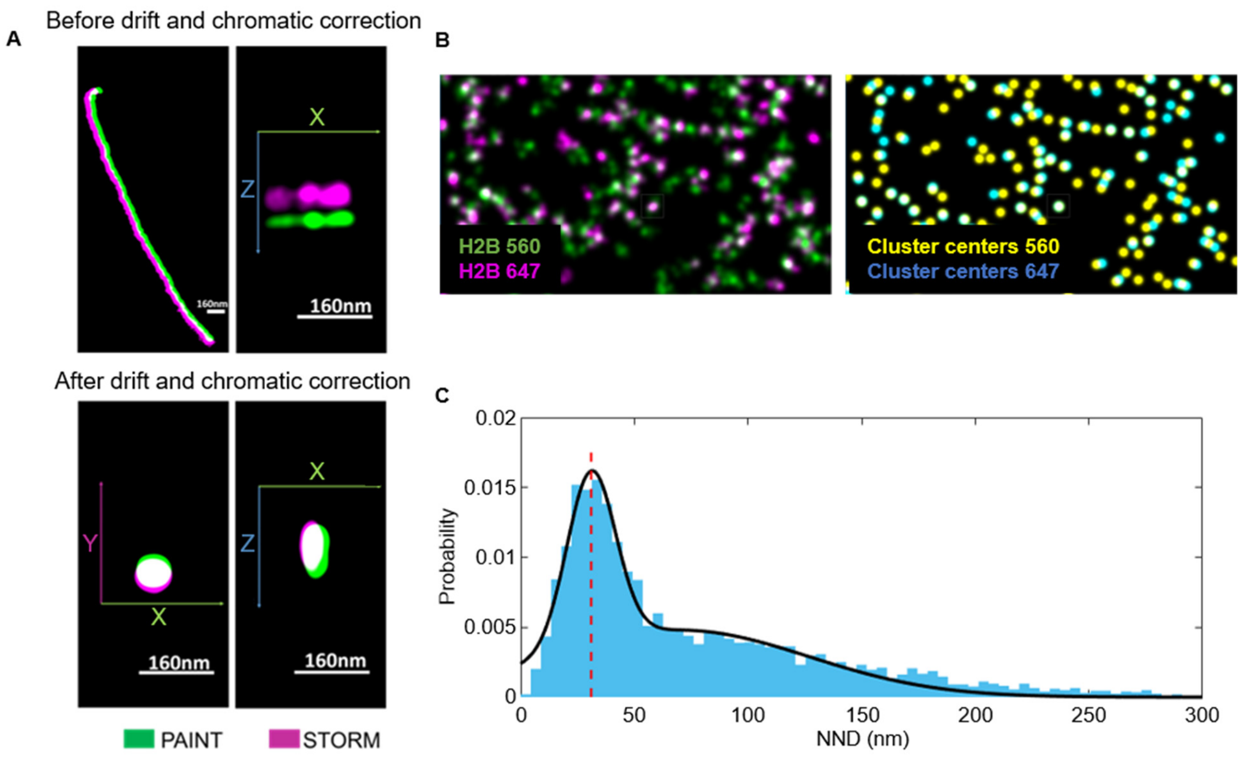

Figure S3. **2-color, 3D DNA, H2B super-resolution imaging workflow and alignment. (A)** A rendered image of the localizations corresponding to a fiduciary bead before (upper) and after (lower) drift correction and 3D alignment. **(B)** H2B was labeled with primary antibodies followed by PAINT-compatible secondary antibodies produced by the Ultivue® Company, which were covalently linked to a single docking strand oligo termed D2. Super-resolution images of H2B were recorded in two-colors using two spectrally separated dyes coupled to the complementary imaging strand (I2) with one dye per oligo. These oligos were termed I2-560 and I2-650 according to the Ultivue® user manual and described as being Cy3- and Cy5-similar, respectively. Both the I2-560 and I2-650 imaging strands were present and imaged simultaneously using 100 ms camera exposure time during acquisition. The resulting image data was aligned (left) using fiduciary beads in the same fashion as other STORM-PAINT data. A large portion of H2B clusters appeared in both color channels (green for I2-560 magenta for I2-650). The H2B clusters were subsequently segmented using the cluster identification algorithm to identify the clutch centers (right). Some segmented clusters only appeared in one color channel likely due to competition between the I2-650 and I2-650 strands for the same D2 docking strand oligo during the finite time of image acquisition.**(C)** The distribution of nearest neighbor distances (NND), calculated between cluster centers in 3D, appeared bi-modal and was fit with a summation of two Gaussian functions (thick, black line). The right-most distribution covering the broad tail corresponds to the clusters appearing in only one color and is centered at 70 nm with a spread of 80 nm. The left-most distribution covering the large peak corresponds to clusters identified in both channels and is centered at 31 nm with a spread of 15 nm. We take this left-most distribution to represent the residual alignment error between DNA and H2B structure in our STORM-PAINT data registration workflow.

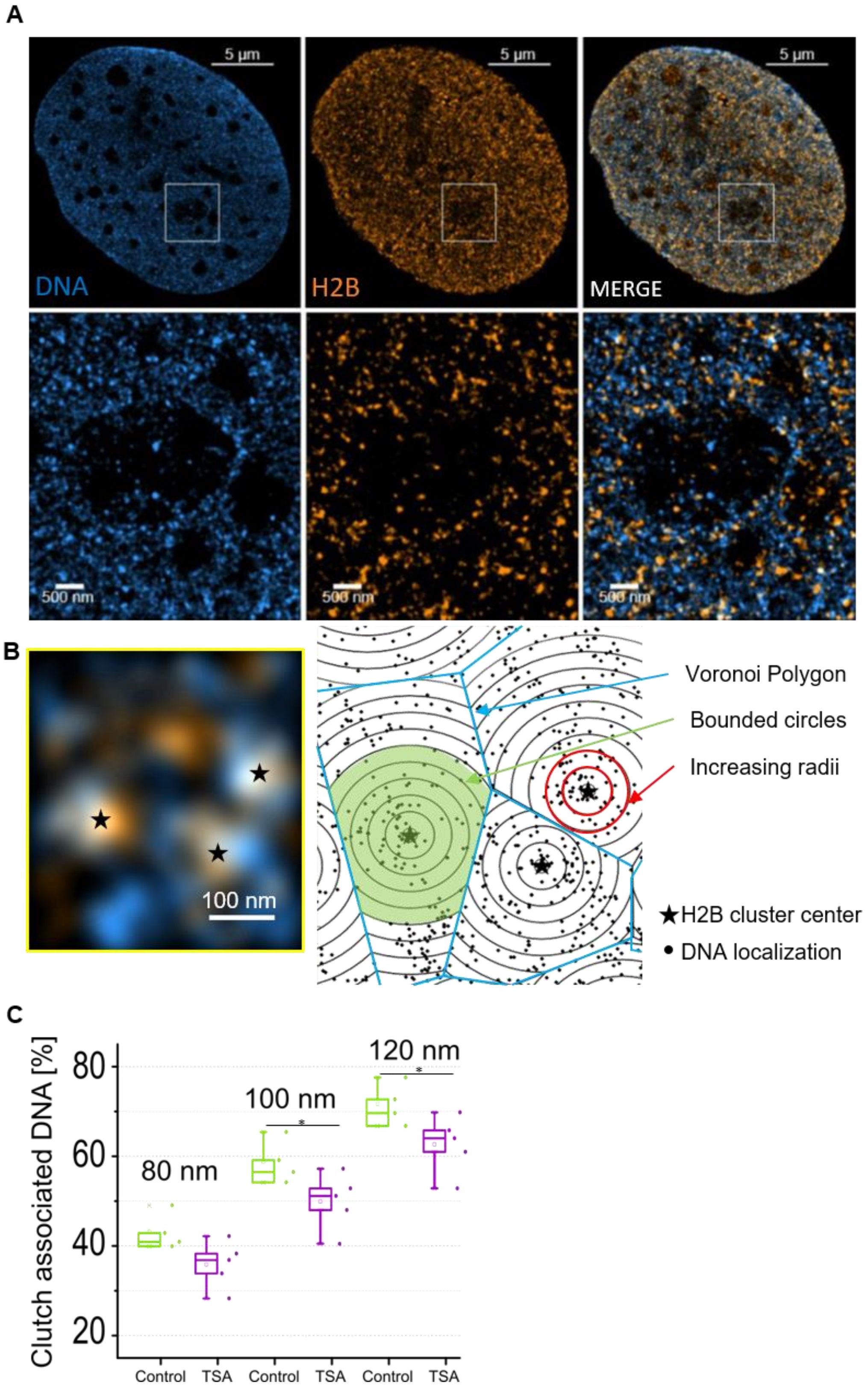

Figure S4. **DNA co-localization with H2B in TSA-treated cells. (A)** Cropped nuclear super-resolution image of EdC-labeled DNA (cyan) and PAINT image of H2B labeled with anti-H2B antibodies (orange) and their overlay in TSA treated human fibroblasts. A zoom of the region inside the white box is shown**. (B)(left)** A representative zoom of a super-resolved region from the cell in (A), **(right)** scheme of the analysis of clutch-bound DNA. The centers of H2B clusters (stars) are the seeds for the

Voronoi polygons (blue) inside which the DNA localizations (black dots) are distributed. Overlaid on top are concentric circles whose radii increase by 10 nm steps **(C)** Percentage of DNA associated to nucleosome clutches in control (green) and TSA-treated (magenta) cells using bound circles with varying search radii. Note: for 80nm, p-value = 0.0635

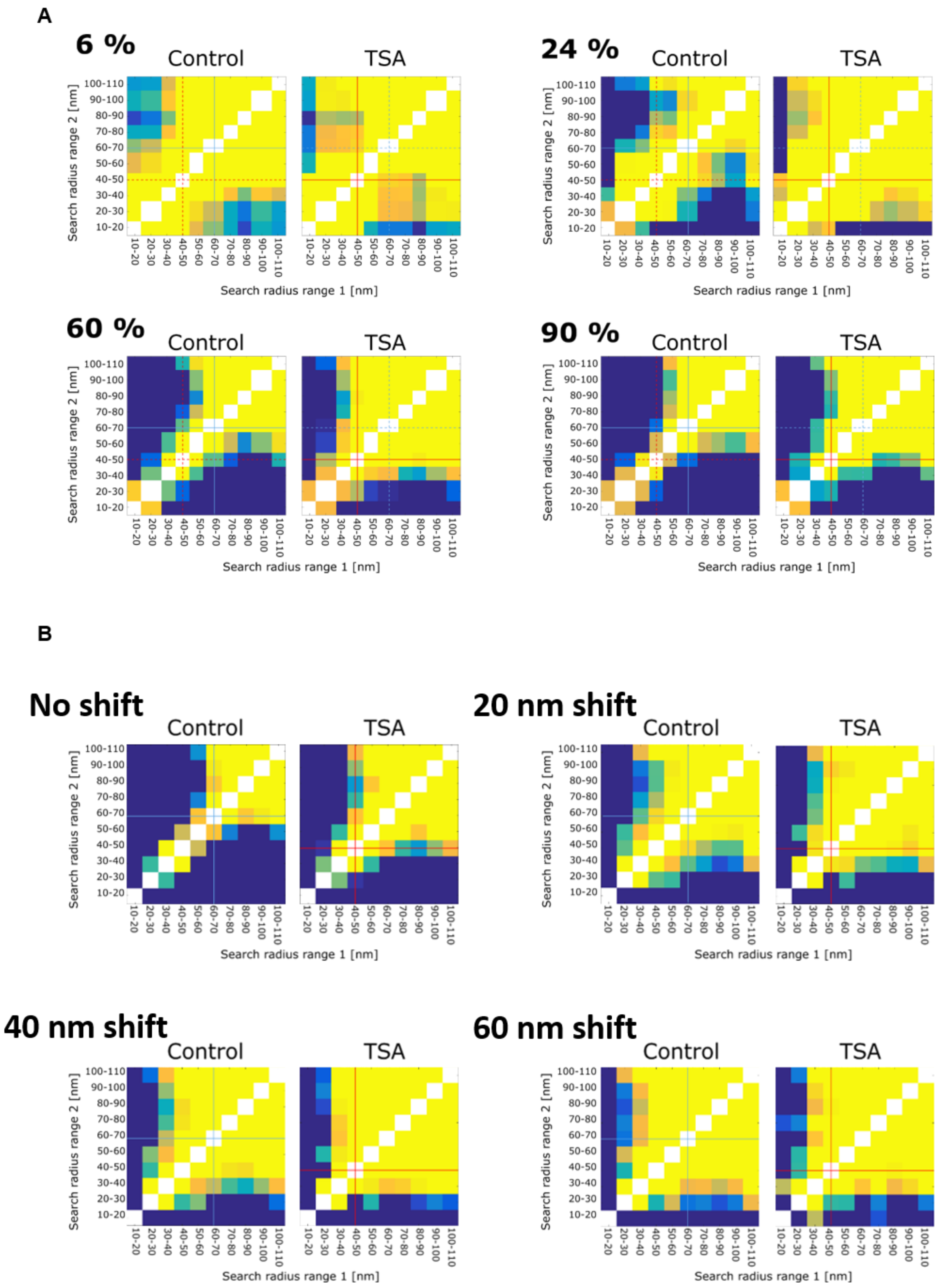

Figure S5. **Similarity matrixes generated from sub-sets of data and shifted data. (A)** Similarity matrix for untreated (left) and TSA-treated (right) cells generated by randomly including different percentages of the available clutch data. The matrices show the level of similarity in DNA density within 10 nm discs of increasing radii. The similarity was calculated as a p-value from Kruskalwallis test and is shown as a color coding corresponding to the color scale bar (from p=0.0001 in blue to p=0.05

in yellow). The diagonal was set to white and not calculated. Cyan and red lines show the boundary of a switch from low to high similarity in untreated and TSA treated cells when calculating the similarity using 100% of data, respectively. The proper trend showing the difference between treated and control cells is apparent only when 60% (corresponding to 1079 clutches/cell in control cells and 904 clutches/cell in TSA-treated cells) or more of the clutch data is included in the analysis. **(B)** Similarity matrix for untreated (left) and TSA-treated (right) cells generated by performing a rigid shift of the DNA data ranging from 20-60 nm before re-performing the radial analysis.

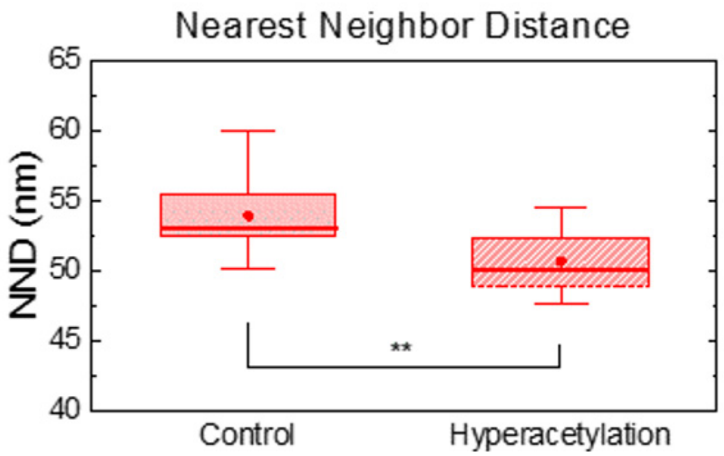

Figure S6. **Inter-clutch NND calculation. (A)** Median Nearest Neighbor Distance measured in nanometers between H2B clutches in untreated and TSA-treated cells. P-value < 0.001

REFERENCES

Allen, G. D. 1990. MODFIT: a pharmacokinetics computer program. *Biopharm Drug Dispos* 11 (6):477-498.

Banterle, N., K. H. Bui, E. A. Lemke, and M. Beck. 2013. Fourier ring correlation as a resolution criterion for super-resolution microscopy. *Journal of Structural Biology* 183 (3):363-367.
